# Syncytiotrophoblast Extracellular Vesicles from Pre-Eclampsia Placentas Differentially Affect Platelet Function

**DOI:** 10.1371/journal.pone.0142538

**Published:** 2015-11-09

**Authors:** Dionne S. Tannetta, Kathryn Hunt, Chris I. Jones, Naomi Davidson, Carmen H. Coxon, David Ferguson, Christopher W. Redman, Jonathan M. Gibbins, Ian L. Sargent, Katherine L. Tucker

**Affiliations:** 1 Nuffield Department of Obstetrics and Gynaecology, University of Oxford, Level 3, Women's Centre, John Radcliffe Hospital, Oxford, United Kingdom; 2 Institute for Cardiovascular and Metabolic Research, School of Biological Sciences, University of Reading, Reading, Berkshire, United Kingdom; 3 School of Physiology and Pharmacology, University of Bristol, Medical Sciences Building, University Walk, Bristol, United Kingdom; 4 Nuffield Department of Clinical Laboratory Science, University of Oxford, John Radcliffe Hospital, Oxford, United Kingdom; 5 Nuffield Department of Primary Care Health Sciences, University of Oxford, Oxford, United Kingdom; VU University Medical Center, NETHERLANDS

## Abstract

Pre-eclampsia (PE) complicates around 3% of all pregnancies and is one of the most common causes of maternal mortality worldwide. The pathophysiology of PE remains unclear however its underlying cause originates from the placenta and manifests as raised blood pressure, proteinuria, vascular or systemic inflammation and hypercoagulation in the mother. Women who develop PE are also at significantly higher risk of subsequently developing cardiovascular (CV) disease. In PE, the failing endoplasmic reticulum, oxidative and inflammatory stressed syncytiotrophoblast layer of the placenta sheds increased numbers of syncytiotrophoblast extracellular vesicles (STBEV) into the maternal circulation. Platelet reactivity, size and concentration are also known to be altered in some women who develop PE, although the underlying reasons for this have not been determined. In this study we show that STBEV from disease free placenta isolated *ex vivo* by dual placental perfusion associate rapidly with platelets. We provide evidence that STBEV isolated from normal placentas cause platelet activation and that this is increased with STBEV from PE pregnancies. Furthermore, treatment of platelets with aspirin, currently prescribed for women at high risk of PE to reduce platelet aggregation, also inhibits STBEV-induced reversible aggregation of washed platelets. Increased platelet reactivity as a result of exposure to PE placenta derived STBEVs correlates with increased thrombotic risk associated with PE. These observations establish a possible direct link between the clotting disturbances of PE and dysfunction of the placenta, as well as the known increased risk of thromboembolism associated with this condition.

## Introduction

Hypertensive disorders in pregnancy including pre-eclampsia (PE) are the commonest causes of maternal mortality worldwide [1]. PE complicates around 3% of all pregnancies, with 10% to 15% of maternal deaths directly linked to PE and eclampsia [2]. The underlying cause remains unclear but it is known to be a placenta driven disorder often associated with poor placental perfusion and the subsequent release of factor(s) that trigger the maternal syndrome of systemic inflammation, vascular endothelial dysfunction and platelet activation, which underlie the symptoms including increased blood pressure, proteinuria, inflammation and hypercoagulation [3, 4]. Women with a diagnosis of hypertension or PE during pregnancy have now been shown to have an increased risk of developing cardiovascular (CV) disease in the long-term, including hypertension (3.7 (2.7–5.05)), ischemic heart disease (2.16 (1.86–2.52)), stroke (1.81 (1.45–2.27)) and venous thromboembolism (1.79 (1.37–2.33) (relative risk (confidence interval))[5]. Earlier detection of PE would present opportunities for more effective clinical intervention with a significant impact on reducing levels of maternal and fetal morbidity, as well as potentially improving long-term outcomes for women affected by PE and their offspring.

Extracellular vesicles (EV) are shed from a variety of cells and have a number of important physiological as well as pathological functions that have attracted increasing attention in vascular research. Indeed, EV originating from platelets, granulocytes, erythrocytes and endothelial cells are associated with a variety of pathophysiological conditions including thrombosis and inflammation. The cell type of origin, cellular activation and micro-environment in which EV are generated is likely to determine their nature, composition and quantity [6]. It is now well established that the placenta releases fetal-derived EVs into the maternal circulation during normal pregnancy, and that this shedding is altered in PE [7–9]. EV are released from the syncytiotrophoblast layer; which covers the surface of the placenta, directly into the maternal bloodstream where they can potentially interact with the endothelium, circulating immune cells and platelets. The release of these syncytiotrophoblast-derived EV (STBEV) in PE is elevated compared to healthy pregnancies, [9–11] and the size, and the profile of proteins present within STBEV in PE, is also significantly altered [9, 12].

It has been reported previously that platelets isolated from women that develop PE in the latter stages of pregnancy display elevated cell surface levels of the platelet activation marker CD63 and exhibit an increase in platelet-bound fibrinogen [13]. Furthermore, PE placentas display a small but significant increase in the expression of the high affinity thrombin receptor PAR1, while women with established PE display elevated STBEV tissue factor activity [14, 15]. It has been hypothesised that these changes may underlie the increased risk of arterial thrombosis during pregnancy and may be relevant to the increased lifetime risk of developing CV disease observed in women who have had PE. It is well established that increased platelet reactivity and/or aberrant platelet activation play a central role in diseases of the CV system, including atherosclerosis, stroke, and myocardial infarction [16]. HELLP syndrome (haemolysis, elevated liver enzymes, low platelet count), a severe complication of PE associated with systemic intravascular coagulation, also points to platelets as a major contributor to disease pathophysiology.

Antiplatelet reagents, most often low dose aspirin, are prescribed to women at high risk of developing PE and have been shown to reduce the relative risk of PE by 17% [17]. We hypothesised that the interactions between STBEV and circulating platelets play a role in the increased platelet reactivity and CV complications associated with PE, during both pregnancy and postpartum. A better understanding of the role of STBEV in platelet activation and the mechanism/s by which platelets are activated in this disease could lead to the development of more targeted therapeutics that selectively inhibit platelet signalling pathways activated in PE, thereby increasing efficacy and decreasing side effects. The aim of this study therefore was to investigate the effect of STBEV, isolated from PE and normal pregnancy derived placentas using dual placental lobe perfusion, on several aspects of platelet function.

## Materials and Methods

### Placenta and Blood Donors and Reagents

Placentas, for isolation of STBEV were obtained from women with normal pregnancies (n = 3) or PE (n = 4) undergoing caesarean section deliveries, within 10 min of delivery and were processed immediately [9]. None of the women were in labour at the time of sampling, and all had singleton pregnancies with no known fetal abnormalities (Table 1). Blood samples for platelet isolation were collected from healthy non-pregnant female volunteers (n = 6) and were processed immediately. These studies were approved by the Oxfordshire Research Ethics Committee C and informed written consent was obtained from all participants.

**Table 1:** Details of the patients who donated placentas.

Patient	Gestation (weeks+days)	Parity	Age (years)	Maximum Systolic (mmHg)	Maximum Diastolic (mmHg)	Protein (dipstick)	Birth Weight (g)
Normal pregnant participants							
N1	38+2	1+1	33	120	70	NAD	3110
N2	41+3	1+2	38	120	78	NAD	4156
N3	38+1	1+0	33	110	50	NAD	3405
**Average**	**39+3**		**34.67**	**120**	**66**		**3557**
Preeclamptic participants							
P1	33+0	2+1	31	205	155	3+	1570
P2	33+3	0+0	30	190	120	2+	1740
P3	36+0	0+1	23	178	100	3+	2495
P4	39+2	1+0	44	150	96	2+	3500
**Average**	**35+4**		**32**	**180.75**	**117.75**		**2326.25**

Note: STBEV preparation P2 was used for immunoblotting and aggregation in response to STBEV and collagen alone. P3 was used for aggregation at 8x10^8^ platelets and for aggregation and activation in aspirinated platelets.

### Isolation of STBEV by *Ex Vivo* Dual Placental Lobe Perfusion

STBEV were isolated using a modified dual placental perfusion system as described previously [9, 18]. Briefly, an individual lobule was isolated and the fetal circulation component perfused with filtered modified M-199 medium containing a bolus of streptokinase (100,000 IU) to promote clot removal, at a rate of 5mL/min. The whole placenta was then laid maternal side up inside a Perspex water jacket maintained at 37°C. The maternal circulation was perfused with 37°C oxygenated media through eight 1.7mm fetal feeding tubes at a controlled rate of 20mL/min. The lobule was perfused for 20min to equilibrate the system, after which time the maternal circuit was closed with a total volume of 600mL perfusion medium. The volume of fetal effluent was measured every 20min and the oxygen concentration of the maternal side perfusate monitored to ensure the stability of the system. Pressure monitors were used to ensure no significant deviations from baseline during the experimental period. At the end of the 3hr perfusion period, the maternal perfusate was centrifuged at 2x1500 xg for 10min at 4°C to remove red blood cells and large cellular debris [19]. The supernatant was then removed and centrifuged at 150,000 xg for 1hr at 4°C to isolate an EV preparation highly enriched for STBEV [9]. The resultant pellets were washed in phosphate-buffered saline (PBS) before being pooled and re-suspended in PBS at a concentration of 5mg/mL protein, determined using a Pierce BCA protein assay kit (Thermo Scientific, Illinois, USA). Aliquots of STBEV were stored at −80°C. Typical STBEV yield was 25–50mg total protein. Flow cytometric analysis of EV produced by this method revealed >90% of vesicles expressed the syncytiotrophoblast specific marker placental alkaline phosphatase (PLAP) and <2% were platelet derived [9]. For aggregation, immunoblotting, thrombus formation and clot retraction assays, three separate STBEV preparations from either normal (N STBEV) or PE (PE STBEV) pregnancies were tested on platelets prepared from at least three donors. Pooled STBEV samples were used for the flow cytometry, electron microscopy and *in vitro* thrombus formation analyses.

### Preparation of Human Platelets

Human platelets were prepared by differential centrifugation as described previously [20]. Briefly, whole blood, drawn into tubes containing acid citrate dextrose (ACD), was centrifuged at 200 xg for 20min at room temperature to obtain platelet-rich-plasma (PRP). For washed platelets, PRP was centrifuged for 10min at 1000 xg in the presence of prostacyclin (PGI_2_) and the cells re-suspended in Tyrodes-HEPES buffer (134mM NaCl, 0.34mM Na_2_HPO_4_, 2.9mM KCL, 12mM NaHCO_3_, 20mM HEPES, 5mM glucose, 1mM MgCl_2_, pH 7.3), to a density of either 4 or 8x10^8^ platelets/mL. Where required, PRP was incubated with 100μM acetylsalicylic acid (aspirin) for 30min at 30°C before being processed as normal to prepare washed platelets [21].

### Flow Cytometric Analysis of STBEV Interaction with Platelets

Platelets were incubated with STBEV that were fluorescently labelled with BODIPY-maleimide (BODIPY FL N-(2-aminoethyl)-maleimide; Life Technologies) to establish whether STBEV interact with platelets. Briefly, a pool of N STBEV (300μL at 5mg/mL of protein) was incubated with BODIPY-maleimide (0.2mM final concentration) for 15min at room temperature before being washed with sterile PBS (2x). Washed platelets at 4×10^8^ cells/mL were then gently mixed with BODIPY-maleimide labelled STBEV (50μg/mL) or Tyrodes-HEPES buffer control at 30°C, then immediately stimulated with 0.5units/mL thrombin or Tyrodes-HEPES control. Stimulation was stopped at 5sec, 90sec, 5min, 20min and 60min by addition of a 4-fold volume of 4% paraformaldehyde in PBS. Fixed platelets were pelleted by centrifugation and resuspended in PBS before processing for flow cytometric analysis. As STBEV are positive for CD41 and CD63 (data not shown), only samples of platelets alone and platelets treated with thrombin were labelled for CD41 (platelet marker) and CD63 (platelet activation marker) to be certain that any positive signal was platelet derived. CD41 positive events were then used to set the platelet gate and surface CD63 expression was used to confirm platelet activation in response to thrombin. Fixed platelets were incubated for 10min at room temperature with Fc receptor block (BD Biosciences, UK), prior to 20min incubation with anti CD41-PECy7 (1ug/mL; Clone P2, Beckman Coulter) and anti CD63-APC (1μg/mL; Clone H5C6, Biolegend) or appropriate isotype controls. Analysis was carried out on a LSR-II flow cytometer (BD Biosciences, UK). A platelet gate was set using CD41 positive events to exclude the smaller unbound STBEV and debris and 10,000 events were collected. The percentage of CD63 and BODIPY-maleimide positive STBEV events in the platelet gate was then recorded as a measure of platelet activation and platelet bound STBEV respectively. Data were analysed using FACS DIVA software (BD Biosciences, UK).

### Transmission Electron Microscopy

To further investigate the nature of the interaction of STBEV with platelets, washed platelets (8x10^8^/mL) were incubated with a pooled sample (n = 3) of N STBEV (50μg/mL) or a Tyrodes-HEPES control. Platelets were then fixed in 4% glutaraldehyde/PBS at 5sec (baseline) and at 1hr time points before centrifuging to pellet the fixed platelets (5000 xg for 5min) and routine processing for transmission electron microscopy (TEM). Samples were post-fixed in 2% osmium tetroxide in 0.1M phosphate buffer, followed by dehydration in ethanol and treatment with propylene oxide prior to embedding in Spurr’s epoxy resin. Thin sections of suitable areas were stained with uranyl acetate and lead citrate before examination using a Jeol 1200EX electron microscope.

### Immunoblotting to Measure Platelet Total Tyrosine Phosphorylation

Washed platelets (n = 3 donors) were incubated with either Tyrodes-HEPES buffer, collagen (0.1μg/mL), N STBEV (n = 3 individual preparations) or PE STBEV (n = 3 individual preparations) for 2min at 30°C. The incubation was stopped with the addition of 3x reducing loading buffer (standard Laemmli recipe [22]) containing inhibitor cocktail. Immunoblotting was then performed as described previously [23]. Briefly, samples were separated on 4–12% gradient SDS-PAGE gels (NuPAGE; Invitrogen) and transferred onto PVDF membrane (Biorad) by semi-dry transfer. Blots were probed for tyrosine phosphorylation using anti-phosphotyrosine antibody (4G10, Upstate (Millipore)) followed by HRP-conjugated secondary antibody (Dako). Bound antibodies were then detected using chemiluminescence substrate (Pierce Biotechnology) and exposure to x-ray film (G.E.Healthcare). Band densities were quantified using Image J software [Abramoff MD, Magelhaes PJ, Ram SJ. Image Processing with Image J. Biophotonics Int 2004;11:36e42], available from http://rsb.info.nih.gov/ij/.

### Measurement of Platelet Aggregation in Response to STBEV

For aggregation assays, platelets were treated with either thrombin (0.5units/mL) or collagen (0.1μg/mL; Nicomed) agonist, or N STBEV or PE STBEV (n = 3) at a final protein concentration of 50μg/mL, using Tyrodes-HEPES buffer as a control, at 30°C with continuous stirring (1200 rpm) in an optical aggregometer. Platelets or PRP, that were pre-treated with STBEV were incubated for 2min prior to the addition of an agonist or Tyrodes-HEPES control. Real time changes in light transmission were logged using a chart recorder for at least 5 min.

### *In Vitro* Thrombus Formation and Clot Retraction Assay

*In vitro* thrombus formation was carried out as described previously [24]. 3,3'-dihexyloxacarbocyanine iodide (DIOC_6_) labelled human citrated blood was pre-incubated for 1min with Tyrodes-HEPES vehicle or with pooled N STBEV or PE STBEV (final protein concentration of 50μg/mL), prior to perfusion over a collagen coated Vena8 Biochip (Cylex Ltd, Ireland) at a shear rate of 20 dynes/cm^2^ at 37°C. Z-stacked images of thrombi were obtained every 30sec for 10min using a Nikon eclipse (TE2000-U) microscope (Nikon instruments, UK). Fluorescence intensity and thrombus volume were calculated using Slidebook5 software (Intelligent Imaging Innovations, USA).

Clot retraction was measured by mixing human PRP (200μl), red blood cells prepared by differential centrifugation (5μl) and STBEV (50μg/mL) from N STBEV (n = 3), PE STBEV (n = 3) or Tyrodes-HEPES buffer alone. Clotting was initiated by thrombin (1U/mL) and a glass capillary placed at the centre of the glass test tube. The weight of the resulting clots was measured after 2hr and 4hr [25].

### Statistical Analysis

Thrombus formation data was analysed by post-hoc multiple comparison testing using a 2-way ANOVA with the Bonferroni correction in PRISM. All other data were analysed by student t-tests. Results are presented as mean values ± standard error of the mean (mean±SEM). P values of <0.05 were considered significant.

## Results

### Platelets and STBEV Associate and the Level of Association Is Increased with Platelet Activation

**Fig 1:**
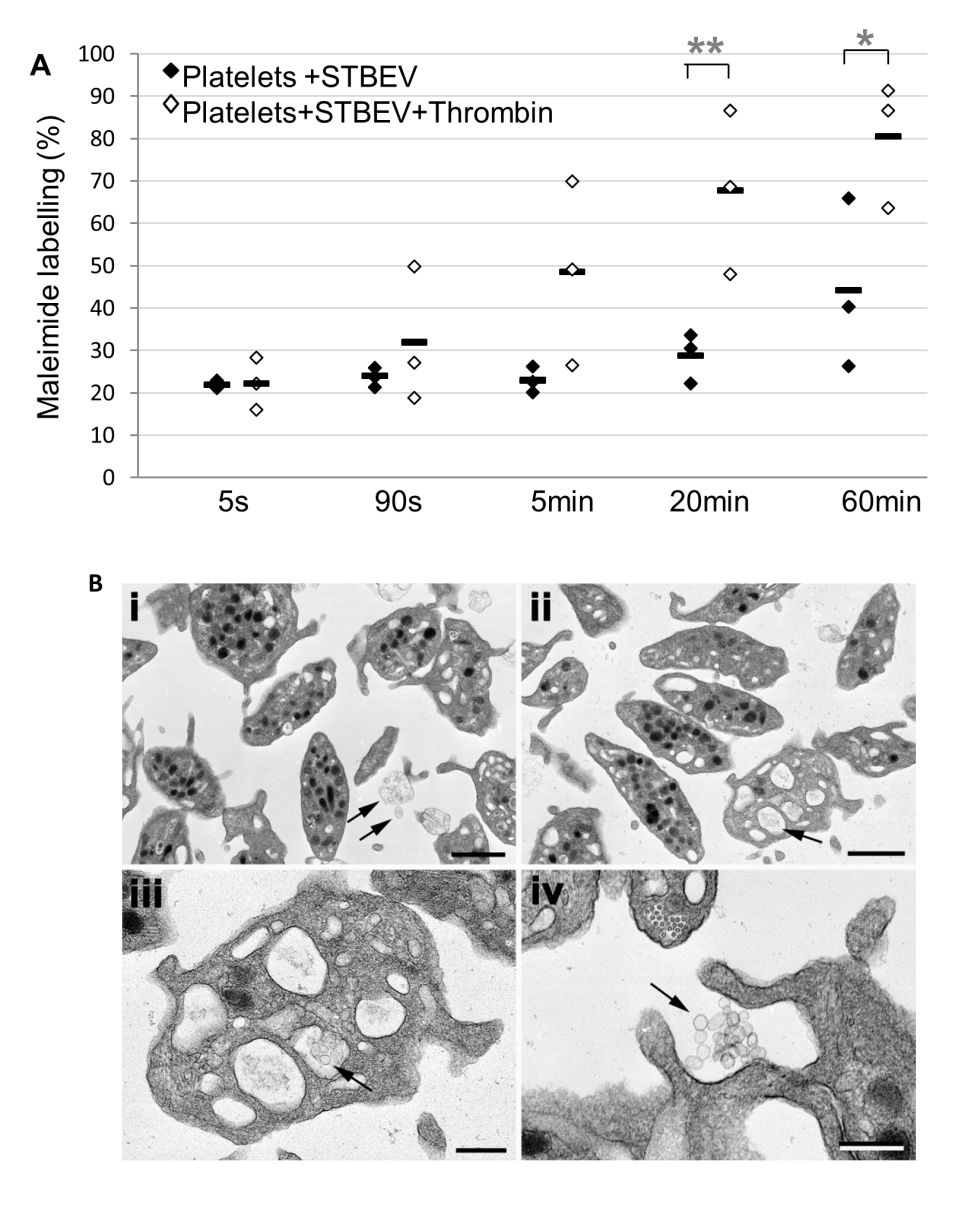
Platelets and STBEV association increases over time and with platelet activation. Washed platelets from 3 healthy donors were mixed with BODIPY maleimide-labelled STBEV in the presence of 0.5units ml/mL thrombin or Tyrodes-HEPES buffer control. Incubation was stopped at 5sec, 90sec, 5min, 20min and 60min followed by labelling for CD41 (platelet marker) and CD63 (platelet activation marker). A platelet gate was set to collect 10,000 events and the percentage of BODIPY maleimide-FITC positive events in the platelet gate recorded. (A) BODIPY maleimide-FITC positive events in the platelet gate showed STBEV binding by platelets that increases over time, with further increases in the presence of thrombin (*p = 0.043, **p = 0.0027). Black bars show mean values. (B) Transmission electron microscopy of washed platelets incubated with STBEV for 5sec (i) and 1hr (ii-iv). The normal ultrastructure of resting platelets can be seen at 5 sec (i).By 1 hour (ii) degranulated platelets were present that contained intra-cellular aggregates of putative STBEV (shown by arrows and enlarged in iii). Phagocytosis of vesicular material was also evident (iv). Bars represent 1μm (i, ii) and 200nm (iii, iv).

Previous reports have suggested that STBEV carry bioactive tissue factor and that platelets internalise tissue factor enriched vesicles [15] [26]. To determine whether STBEV obtained by *ex vivo* dual placental perfusion associate with platelets (either by surface binding or uptake), washed platelets were incubated with BODIPY-maleimide labelled STBEV and samples taken at different time points and analysed by flow cytometry. The proportion of BODIPY-maleimide positive platelets increased from 21.9% (±0.6 S.E.) after 5sec (baseline) to 44.2% (±14.2 S.E.) after 60min (Fig 1A). No increase in fluorescence was detected when using BODIPY-maleimide labelled STBEV alone, eliminating the possibility that fluorescent STBEV aggregates were contributing to the signal from the platelet gate. Treatment with thrombin (0.5units/mL) resulted in an additional increase in BODIPY-maleimide labelled STBEV positive events when compared to resting platelets (from 22.2% (±4.3 S.E) at baseline to 48.5% (±15.3 S.E) at 5min and 80.5% (±10.5 S.E.) after 60min (p = 0.0027 compared to resting at 60min)) (Fig 1A).

To investigate whether STBEV associate with the surface of platelets or are internalised, electron microscopy was carried out on resting platelets, and following incubation with STBEV for 5sec and 1hr (Fig 1B). The resulting images suggest that STBEV rapidly associate with platelets and are internalised over time, with concomitant degranulation (Fig 1C–1E).

### Platelet Activation Is Increased by Incubation with STBEV

**Fig 2:**
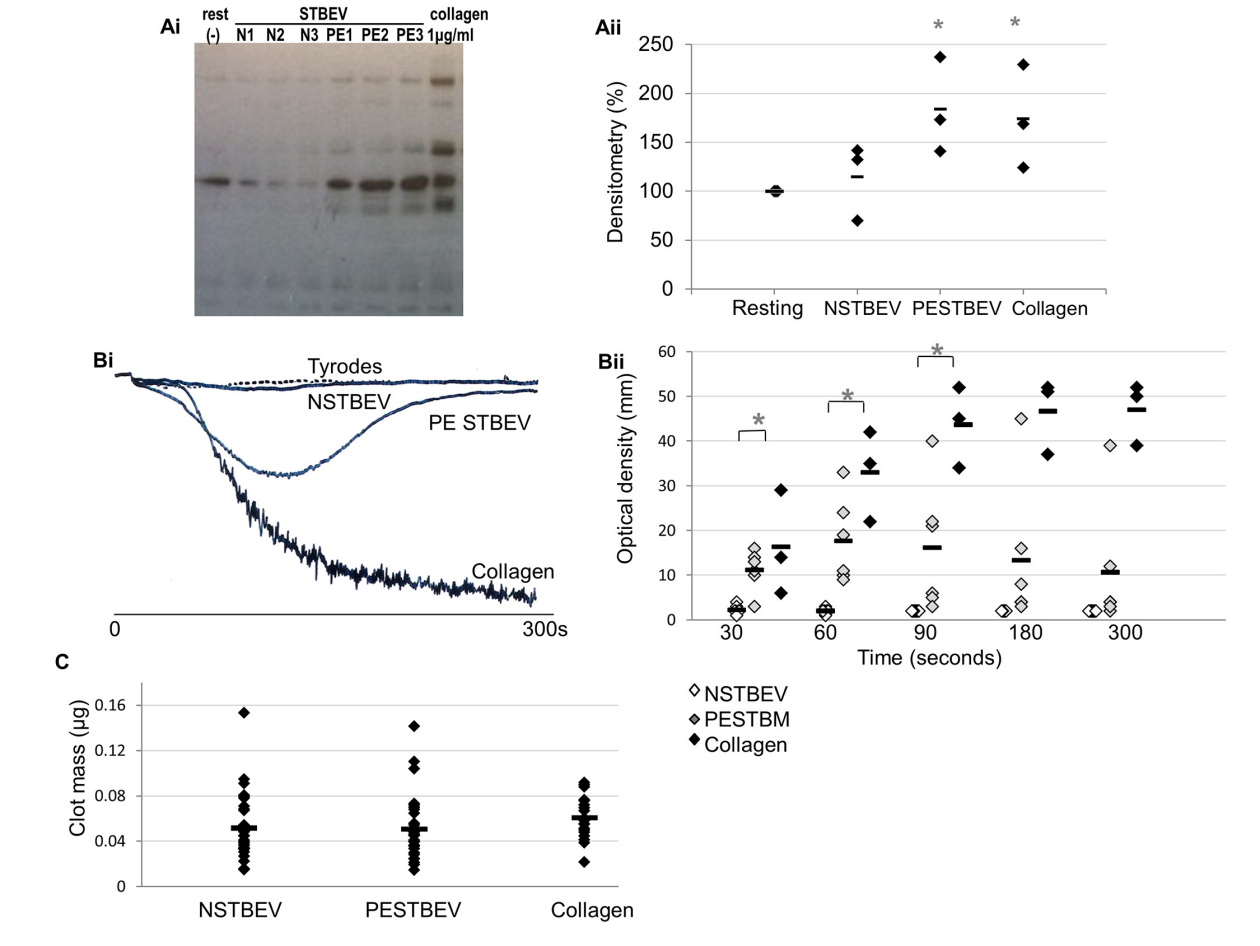
Platelet activation is increased by incubation with PE STBEV and aggregation and clot retraction are affected. (Ai and ii) Washed platelets (n = 3 donors) were exposed to placental perfusion derived STBEV from healthy (N STBEV; n = 3) and pre-eclamptic (PE STBEV) pregnancies for 90sec. Samples were separated by SDS-PAGE and immunoblotted for tyrosine phosphorylation. (Bi (representative experiment) and ii) Aggregation of washed human platelets (n = 3) at 8x10^8^/ml was measured in the presence of collagen (0.1μg/ml), N STBEV (n = 3) or PE STBEV (n = 3) over 5min. (C) Clot retraction (n = 11 blood donors) in the presence of N STBEV (3 different placental donors), PE STBEV (3 different placental donors) or a Tyrodes-HEPES control. Clots were weighed after 2hr. Black bars show mean values and * p< 0.05.

To determine the effect of N STBEV and PE STBEV on platelet activation, total tyrosine phosphorylation in washed platelets incubated with normal or PE STBEV was examined. A significant increase in platelet tyrosine phosphorylation was seen in response to PE STBEV (p<0.05), the protein profile is similar to that caused by collagen stimulation although the intensity of specific bands is altered (Fig 2A). No effect of N STBEV on platelet tyrosine phosphorylation was evident (Fig 2A).

### STBEV Cause Reversible Aggregation at High Platelet Concentrations

Previous reports had shown that PE STBEV have higher surface expression of tissue factor and placental tissue factor causes platelet activation at high platelet concentrations [15] [26]. To assess the response of platelets to STBEV produced by the dual perfusion method, aggregation assays were performed in the presence and absence of STBEV using platelets at a concentration of 8x10^8^ /mL. Platelets did not aggregate when incubated with N STBEV, but reversible aggregation was induced by PE STBEV (Fig 2B). Visual inspection of the samples following the assay confirmed that no large visible aggregates remained. Aggregation was not observed in response to STBEV at a platelet concentration of 4×10^8^/mL (data not shown). This is typical of weak platelet stimulation in which substantial levels of secretion are not observed.

### Normal and PE STBEV Affect Rate of Clot Retraction

The latter stages of thrombus formation involve clot retraction which is driven by a contraction of the platelet cytoskeleton and extracellular matrix and is co-ordinated through integrin *αIIbβ3* signalling. Incubation with N STBEV and PE STBEV caused a small but significant decrease in clot size at 2hr (p<0.05; Fig 2C) but this effect was no longer apparent by 4hr, with no significant difference between N STBEV or PE STBEV and Tyrodes-HEPES buffer control.

### Agonist Induced Aggregation Is Attenuated by Incubation with STBEV

**Fig 3:**
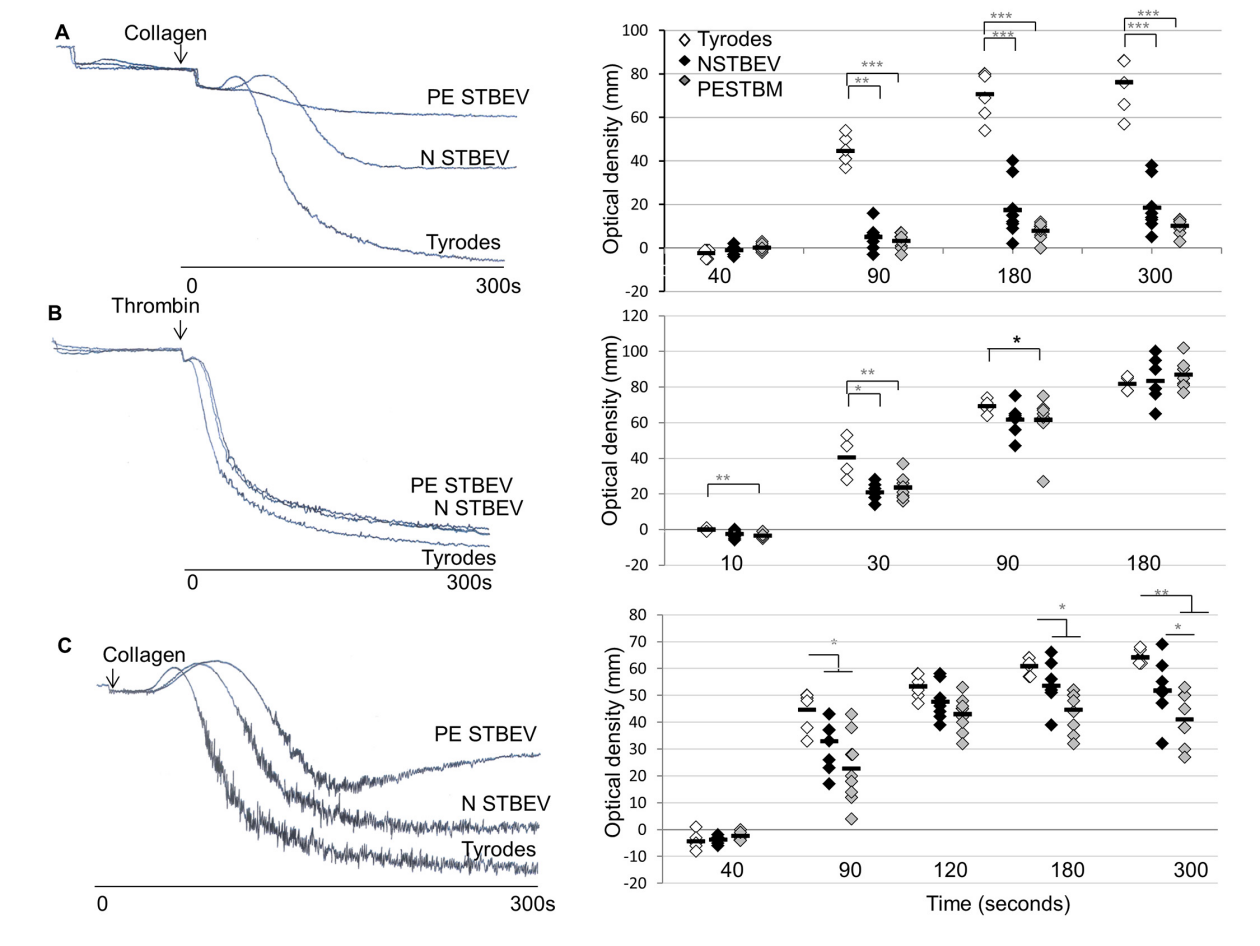
STBEV attenuate platelet aggregation in response to collagen and thrombin. Representative aggregation traces and combined percentage aggregation plots of (A) washed platelets preincubated with N STBEV, PE STBEV or Tyrodes-HEPES buffer (2 min) then stimulated with collagen (final concentration 1μg/mL), (B) washed platelets preincubated with N STBEV, PE STBEV or Tyrodes-HEPES buffer then challenged with thrombin and (C) Platelet rich plasma preincubated with N STBEV, PE STBEV or Tyrodes-HEPES buffer and then stimulated with collagen at a final concentration of 1 μg/mL. In each case aggregation was followed for 5 min and the distance travelled on the chart recorder (mm) plotted from 3 replicate experiments where each platelet preparation was exposed to 3 different PE STBEV and N STBEV preparations. Black bars represent mean values and *P < 0.05.

The effect of STBEV pre-treatment on agonist-induced aggregation was examined in washed platelets (4×10^8^/mL) and platelet rich plasma (PRP). A significant decrease in platelet aggregation, in response to collagen, was observed at 90sec, 3min and 5min for both washed platelets and PRP following pre-treatment (2 min) with STBEV (Fig 3A and 3C). The observed decrease was significantly greater in response to PE STBEV compared to N STBEV (Fig 3A and 3C) (P≤0.05 at 5min following addition of collagen). The presence of plasma components such as the clotting factors did not rescue aggregation (Fig 3C). A similar response to collagen was seen following incubation with STBEV for 20min (data not shown) and a similar, but much less pronounced trend was seen with thrombin treatment (0.5units/ml)(Fig 3B). This suggests that this could be agonist specific effect though a full dose response would be required to determine this.

### Aspirin Abrogates STBEV-Induced Aggregation

**Fig 4:**
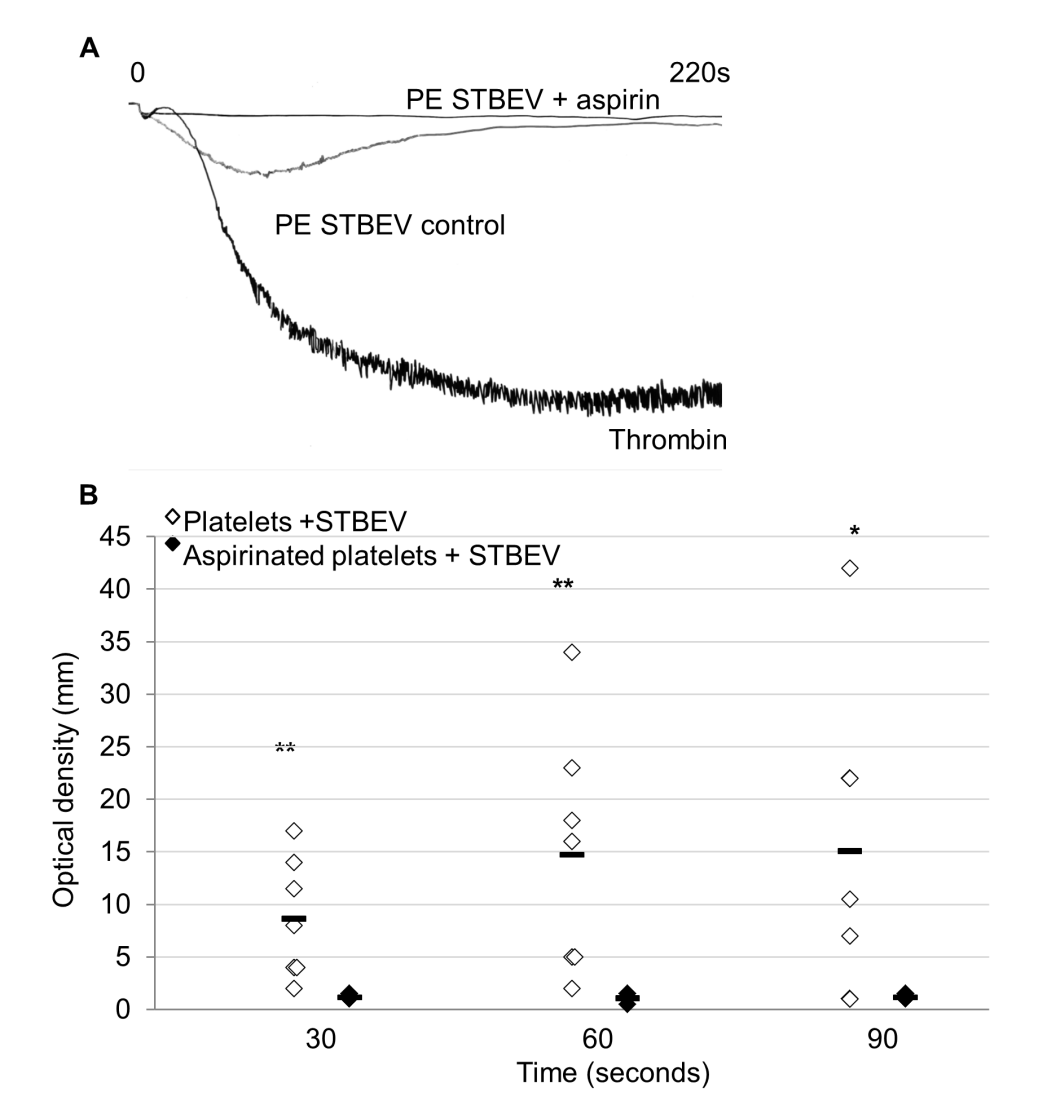
Aspirin treatment abolishes platelet aggregation in response to PE STBEV. Platelet rich plasma was incubated with 100 μM acetylsalicylic acid (aspirin) or DMSO control for 30 minutes at 37°C. Platelets were then isolated and stimulated with thrombin, or PE STBEV (final concentration of 50μg/mL) at 37°C with continuous stirring (1200 rpm) in an optical aggregometer. Aggregation was observed over 5 min. (A) Representative aggregation traces and (B) plot of aggregation replicates (n = 3 individual platelet preparations) * P≤0.05 **≤0.01

Prophylactic aspirin has been shown to reduce the relative risk of PE [17]. To investigate the effect of aspirin on the PE STBEV-induced changes to aggregation observed above, PE STBEV were added to aspirin-treated or non-aspirin-treated washed human platelets at 8x10^8^/mL. Aspirin treatment completely blocked PE STBEV-induced aggregation (Fig 4).

### Thrombus Formation Increases with STBEV

**Fig 5:**
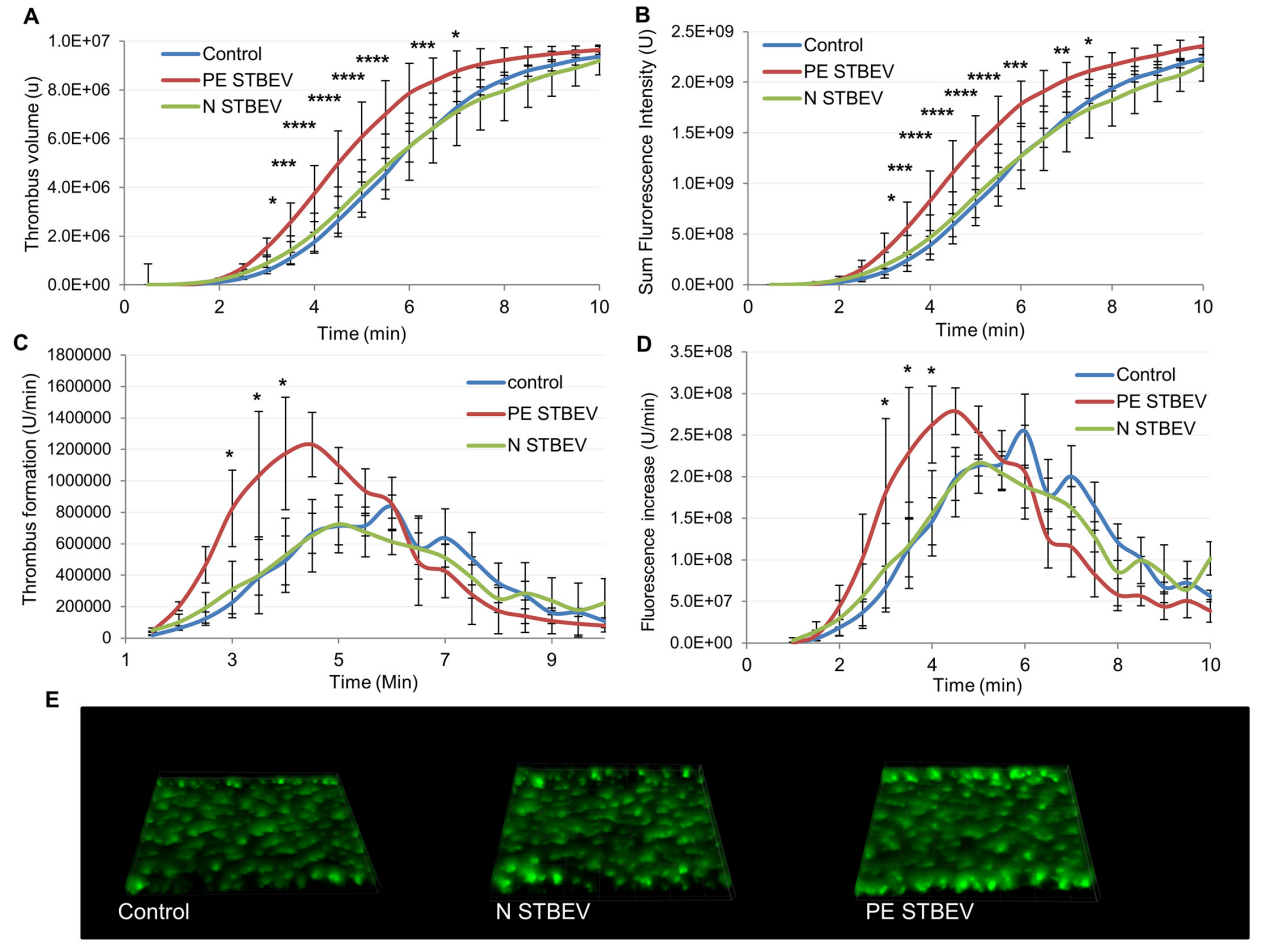
STBEV increase thrombus formation. Whole blood mixed with the lipophilic dye 3,3-dihexyloxacarbocyanine iodide was treated with N STBEV, PE STBEV or Tyrodes-HEPES control and perfused through collagen-coated (400 μg/mL) Vena8Biochip at a shear rate of 20 dyn/cm2. Thrombi were recorded through a series of images in the Z-plane through their full depth every 30sec using a Nikon eclipse (TE2000-U) microscope, thrombus fluorescence intensity was calculated using Slidebook, Version 5. The data represent thrombus volume (A) and sum fluorescence (B) (total fluorescence) intensities and are the mean of 3 separate experiments on different blood donors. A post-hoc 2-way ANOVA with Bonferroni correction showed no significant difference between control and N STB EV, the rate of thrombus formation (rate of change) (C and D) was increased in PE STBEV vs control (*P≤0.05, ***P≤0.001, ****P≤0.0001) though there was no difference in the final thrombus size at 10 min. (E) 3D rendered images of thrombi formed under each experimental condition.

To assess the effects of STBEV on platelet activity under more physiologically relevant conditions, thrombus formation in response to collagen was measured under flow. Overall, an increase in the rate of thrombus formation was observed in the presence of STBEV with PE STBEV showing a significantly increased rate of thrombus formation. No effect was seen in response to the addition of N STBEV (Fig 5).

## Discussion

Previous work has shown that platelet reactivity is increased in women who develop PE [13] and that the placenta sheds increased amounts of STBEV, with altered phenotype and cargo, into the maternal circulation during PE [10, 11]. Our aim was to provide a better understanding of the role, if any, of STBEV in PE-associated changes in platelet reactivity.

This study shows that STBEV interact with platelets and affect their function. Flow cytometry revealed an interaction between platelets and STBEV that increased when platelets were activated with thrombin, suggesting low levels of STBEV may bind platelets in normal pregnancy, whereas in the pro-coagulant environment of PE, there may be a greater affinity of activated platelets for STBEV. Electron microscopy suggested a rapid association between STBEV and platelets followed by internalisation within 1hr. This is in line with previous work showing that tissue factor-enriched vesicles prepared commercially from placenta are rapidly taken up by platelets [26]. Activation was also suggested in the TEM images by the degranulation of platelets treated with STBEV.

Examination of the effect of STBEV on global tyrosine phosphorylation and platelet aggregation showed that exposure to PE STBEV increased platelet activation. No effect on activation was seen in response to N STBEV. Platelets and STBEV are considered to be ‘sticky’; a property that could, in theory, explain some of the observations. However, the clear differences in platelet response to STBEV from healthy and PE pregnancies strongly suggest that this is a mechanistic effect rather than a simple association between ‘sticky’ entities. Interestingly, and in agreement with previous findings [26], at high platelet concentrations, STBEV cause spontaneous reversible platelet aggregation, an effect that was dependent on platelet concentration (8x10^8^/ml) and unique to PE STBEV. The mechanism/s involved are currently under investigation, as is the suitability of the *in vitro* aggregation system, where exposure to STBEV is acute and components can become rate limiting, to model the *in-vivo* situation where active constituents can be continually replenished and exposure to STBEV is over a much longer time frame. Clot retraction in the presence of N STBEV and PE STBEV revealed a small overall increase in the rate of clot retraction at 2hrs, suggesting that N STBEV and PE STBEV accelerate, but do not enhance or inhibit, overall clot retraction to the same degree.

To determine whether the presence of STBEV affects normal platelet responses to known agonists, washed platelets and platelet-rich plasma were challenged with collagen or thrombin following pre-treatment with STBEV. Aggregation was reduced in both the washed platelets and PRP in response to collagen and thrombin. Although it is possible that STBEV can sterically inhibit aggregation, the fact that PE STBEV have a greater inhibitory effect than those from normal placentas again suggests a qualitative effect, rather than STBEV just physically blocking platelet-platelet interactions. This is in line with previous work that showed ADP-stimulated aggregation was reduced in response to incubation with placental brush border membrane vesicles, potentially due to an inhibition of thromboxane A_2_ [27, 28]. A decrease in agonist-induced platelet aggregation appears contradictory to our observations that STBEV promote activation and spontaneous aggregation. Currently we can only speculate on possible mechanisms underlying this observation, such as the initial activation and internalisation of STBEV potentially altering platelet plasma membrane composition or receptor trafficking such that platelets are desensitised to subsequent challenges.

We found that aspirin (100 μM), at concentrations similar to that in patients on standard doses (30–100 mg/L equivalent to 200–600 μM), abolished the aggregation effects caused by STBEV interaction with platelets *in vitro*. This suggests that aspirin may confer some of its clinical benefit by blocking STBEV driven platelet aggregation *in vivo*. Aspirin treatment is known to reduce the effect of platelet agonists through the inhibition of cyclooxygenase 1 activity and subsequent thromboxane A_2_ generation [29]. The observation that aspirin inhibits PE STBEV stimulated aggregation suggests that these EV require activation of platelet signalling to promote aggregation, and do not simply agglutinate platelets. This may, in part, explain the mechanism by which low-dose aspirin reduces the CV complications and development of PE. The involvement of the second messenger thromboxane A_2_ in STBEV-mediated effects and the potential alterations in cell surface composition following STBEV exposure will require further investigation.

Thrombus formation was investigated *in vitro*. Our results show that PE STBEV increase the rate of thrombus formation in whole blood under flow conditions (P≤0.05 at 3–4 minutes) therefore the size of thrombus formed is also increased (P≤ 0.05 at 3–6.5 minutes). There was a high degree of variability between donors not usually seen with this technique, which may indicate some individuals are more susceptible than others to the effects of STBEV, and may be in line with the variability of pathology in PE patients.

The concentration of STBEV used here was 50μg/ml, which is higher than previously found in the maternal bloodstream (e.g. 26 ng/mL in healthy pregnancy, 42 ng/mL in late onset PE and 71 ng/mL in early onset forms of the disease [30] however, it is likely that local concentrations will vary, especially at areas of STBEV release and reduced flow in the intervillous space of the placenta. STBEV have been detected in the circulation as early as the first trimester [10, 31]. In general, STBEV from patients with PE had a greater effect on platelet function and activation, suggesting that STBEV could contribute to the thrombotic pathology of PE. If a causative role for STBEV in PE can be established, and relevant activation pathways identified, this would facilitate interventions to block platelet-STBEV binding, or the affected pathways to be targeted. It should be noted that the size and rate of release of STBEV is increased in PE [7, 9], however for this work STBEV samples were normalised for protein concentration. Therefore, the difference between healthy pregnancy and PE *in vivo* may be even more pronounced.

Microvesicles and exosomes are produced via distinct mechanisms from the STB and have different compositions and functions [32]. Exosomes are produced in the endosomal compartment through the production of multivesicular bodies and are constitutively secreted by the STB [8, 11]. They possess a distinct profile of proteins including pro-apoptotic proteins and TGFβ and are immunosuppressive [33]. Microvesicles on the other hand are pro-inflammatory and may have beneficial local effects during the early stages of normal pregnancy by increasing STB exosome production, as cellular stress has been shown to drive exosome production in other cell types [34]. It has previously been demonstrated that there is a higher level of EV production in PE and that the vesicles produced are larger in size [7–9]. During healthy pregnancy, a ratio between microvesicles and exosomes may be maintained that allows for sustained low-level inflammation that drives exosome production. If the ratio were disturbed, by poor placental perfusion and the resultant trophoblast oxidative and ER stress, this could lead to the excessive production of pro-inflammatory, procoagulant and anti-angiogenic microvesicles, effects that are central to the pathology of PE. Perturbation of the microvesicle-exosome ratio and difference in STBEV composition may also explain the heterogeneity in maternal symptoms and could provide an explanation for the divergent effects on platelets of N STBEV and PE STBEV described here.

This work has provided new insight into the role of STBEV in the platelet dysfunction associated with the multifactorial and multisystem condition of PE.
